# Books and Magazines

**Published:** 1911-03

**Authors:** 


### Books and Magazines.

CASE HISTORIES IN PEDIATRICS. By Jno. Lovett Morse, A. M., M. D., Assistant Professor of Pediatrics, Harvard Medical School; Associate Visiting Physician at Infants' Hospital and at Children's Hospital, Boston. Cloth; 320 pages. Illustrated. W. M. Leonard, Publisher, Boston. Price, $3.00.

This book presents a post-graduate clinical course in diseases of children.

The author has selected one hundred actual case histories, classified them under section headings, "Diseases of Nutrition," etc., and considered each case under the captions History, Physical Examination, Diagnosis, Prognosis and Treatment exactly as he has done before his post-graduate classes.

A very complete index makes the book valuable for systematic reference.

There is a vitality in this kind of book that is refreshing. It deals with actual conditions as the doctor meets them. It is absolutely true throughout, without possible error or misstatement, for these are actual cases. There is not a superfluous line in the book.

It is a book which the doctor will read for a short or longer time with interest and to which he will look for helpful suggestion as to a consultant and not be disappointed.

We know of no more valuable book upon this important subject published in recent years for the doctor's actual use.

---

WYETH'S SURGERY. Revised, rewritten and thoroughly up to date.

NEW YORK CITY, 1911.

DEAR DOCTOR: The original "Text-Book on Surgery," by this author, was published in 1887, and was followed by two subsequent editions, the last of which appeared in 1900. These editions were published and widely distributed by D. Appleton & Co., from whom, by an arrangement mutually satisfactory, the illustrations and copyright were obtained.

In the new book, "Wyeth's Surgery," a single volume of over 828 pages, all the illustrations relating to modern technic which appeared in the original publications, and as much of the text as deals with the science and art of surgery as accepted and practiced at this date, have been retained, while many new illustrations have been added, making 864 in all, of which 57 are colored.

No expense has been spared in the production of this volume, which is printed on the heaviest and best paper and neatly bound in Bancroft linen. It will be sent by post or express to any point in the United States and Canada upon receipt of three dollars and sixty cents ($3.60). The amount may be sent preferably by post-office order or New York exchange.

Very truly yours,

A. I. WOOD,

244 Lexington Ave., New York City.

In speaking of this book, a recent interviewer says: "'Wyeth's Surgery' reflects great credit not only upon its author, but upon American surgery. The text is written in a very interesting and instructive manner, and the subject matter has been selected with care and brought well up to date, presenting in one handsome volume and in compact form the status of American surgery of today.

"Some of the strong points in the author's character—originality in research, honesty of purpose, carefully guarded statements, a tender and watchful care for the interest of his patient, and the helpful, willing spirit with which he always meets those who seek his counsel and advice—are vividly portrayed on every page of this admirable work. The book might well be called Surgical Sermons by John A. Wyeth.

"The author's well-known, simple technic and his careful attention to details, are everywhere apparent, and are a prominent feature of the work. To the young surgeon, the conservative teaching of this book will be found a safe and reliable guide, and will aid materially in making for him a reputation as a good surgeon. In the issue of this book, the author has placed the medical profession under an obligation to him that it can never fully repay.

"No American surgeon is better known or has more friends than Dr. John A. Wyeth, and by all his book will be eagerly sought and read, bringing relief to the patient and comfort and profit to the surgeon. This volume should be in the hands of every surgeon, general practitioner and medical student. No medical man who wishes to keep abreast of the times, whether he be engaged in city or remote country practice, can afford to do without 'Wyeth's Surgery.'"

